# The nanoemulsion adjuvant provides antigen dose-sparing effects and enhances maternal passive immune protection for the cell-cultured quadrivalent influenza virus subunit vaccine

**DOI:** 10.1186/s12985-025-03006-z

**Published:** 2026-01-30

**Authors:** Yutian Wang, Zheng Jia, Ying Liu, Shuang Li, Yongbo Qiao, Dapeng Zhao, Jianyang Gu, Yehong Wu

**Affiliations:** 1 Changchun Institute of Biological Products Co.,Ltd., Changchun, 130012 Jilin Province China; 2 State Key Laboratory of Novel Vaccines for Emerging Infectious Diseases, National Biotec Group Company Limited, Beijing, 100024 China

**Keywords:** Nano emulsion adjuvant, Quadrivalent influenza vaccine, Subunit vaccine, Dose-sparing, Immune effect, Challenge protection

## Abstract

To elicit a robust immune response, an adjuvant can be combined with the antigen in influenza vaccine formulations. In this study, we evaluated the dose-sparing effect and safety of a squalene-based oil-in-water nanoemulsion (NE) adjuvant formulated with a cell culture-derived quadrivalent influenza vaccine. Immune responses-including anti-HA IgG antibody levels and hemagglutination inhibition (HAI) titers-were assessed, along with protection against a homologous challenge with influenza B virus (strain B/Maryland/15/2016 B/Victoria). We also investigated the influence of antigen dose on vaccine-induced immunity and the passive protection conferred to offspring via maternal antibody transfer. The NE adjuvant elicited strong anti-HA antibody responses in young adult mice, and these antibodies were effectively transferred from immunized mothers to their offspring. Furthermore, offspring born to NE-immunized mothers were protected against influenza virus challenge. Collectively, our results indicate that the NE formulation induces potent influenza-specific immune responses with dose-sparing effects and enables maternal transfer of protective immunity. These findings support the potential of NE as an effective adjuvant for MDCK cell-based influenza subunit vaccines. Importance. This study demonstrates that a squalene-based nanoemulsion (NE) adjuvant significantly enhances the immunogenicity and dose-sparing capacity of cell culture-derived quadrivalent influenza vaccines. Key advances include: (1) NE-adjuvanted vaccines achieved a remarkable 125-fold antigen dose reduction while maintaining antibody titers comparable to high-dose formulations, addressing critical challenges in pandemic preparedness; (2) NE induced robust humoral and cellular immunity, including elevated anti-HA IgG (10-fold increase vs. non-adjuvanted vaccine), improved HI titers, and enhanced IFN-γ/CD8 + T-cell responses; (3) Unique maternal-offspring protection was demonstrated, with transferred maternal antibodies conferring 100% survival in offspring against viral challenge. These findings position NE as an effective adjuvant technology that simultaneously optimizes antigen use and broadens immune protection across age groups.

**Supplementary Information:**

The online version contains supplementary material available at 10.1186/s12985-025-03006-z.

## Introduction

Influenza, an acute respiratory illness caused by influenza viruses, represents a persistent global health threat. Its status as a leading infectious disease necessitates continued prioritization in public health control strategies. Vaccination serves as the most effective preventive measure against influenza viruses, with most commercially available vaccines currently being produced through the embryonated egg-based manufacturing process [1]. Although the technology for egg-based influenza vaccines is mature and cost-effective, it still has several limitations. Large-scale production requires an adequate supply of eggs, and production needs to start long before the influenza season begins, which limits the ability of egg-based vaccines to respond to new viral strains. Most importantly, human influenza viruses undergo mutations in the hemagglutinin protein to facilitate optimal growth in embryonated eggs [2, 3]. These mutations may lead to antigenic mismatch between vaccine strains and circulating strains, thereby compromising vaccine efficacy [4, 5]. Furthermore, vaccine effectiveness in preventing influenza-related illnesses demonstrates variable protection levels (40–60% efficacy) due to multiple contributing factors: the antigenic drift of influenza viruses, age-related or comorbidity-associated mild immunodeficiency, and immune imprinting effects from prior similar influenza exposures [6, 7].

Over the past three decades, complementary to the annual WHO-recommended vaccine strain updates, multifaceted strategies have been implemented to address the limitations of conventional seasonal influenza vaccines [6, 8]. These strategies include alternative administration routes such as intradermal injection to enhance immunogenicity, although this method requires strict hygiene conditions and is associated with increased adverse reactions. While increasing the antigen content in vaccines to enhance efficacy has been attempted, this approach has shown limited effectiveness while significantly raising production costs. The addition of adjuvants and the development of novel production processes have enabled the cultivation of vaccine antigens in mammalian cells, thereby circumventing avian adaptation mutations during production and improving the match between vaccine strains and circulating viruses [9–11]. Collectively, these advancements have established cell-based subunit vaccines containing safe and effective adjuvants as a more advantageous solution for addressing influenza pandemics.

Cell-based manufacturing offers improved growth of influenza viruses and better matching with circulating viral strains, potentially resulting in higher protective efficacy [4, 12]. These adjuvanted influenza vaccines, being devoid of egg-derived proteins, eliminate the risk of egg-associated hypersensitivity reactions in susceptible populations [1, 3]. In preclinical mouse studies, it has been shown that adjuvanted influenza vaccines exhibit comparable safety profiles to conventional non-adjuvanted formulations while conferring superior effectiveness through multiple mechanisms [13–15]. The cell-based quadrivalent influenza vaccine Flucelvax Tetra^®^ has received regulatory approval in both the European Union and United States for intramuscular administration in individuals ≥ 6 months of age. Although the egg-derived adjuvanted quadrivalent vaccine Fluad Quadrivalent^®^ is licensed for geriatric populations (≥ 65 years), no market-authorized adjuvanted quadrivalent subunit influenza vaccine currently exists that combines cell-culture production with adjuvant technology [1].

In this study, BALB/c mice were immunized with a cell-based quadrivalent subunit influenza vaccine (QIVc) alone or in combination with a squalene-based nanoemulsion adjuvant (NE). Serum IgG titers, hemagglutination inhibition (HAI) titers, the number of IFN-γ-secreting immune cells, and CD4/CD8 splenocyte populations were measured. The protective efficacy of the vaccine was evaluated through challenge experiments using influenza virus (B/Maryland/15/2016). Additionally, the impact of vaccine dose on HA-specific immune responses and the protection conferred to pups via maternal antibody transfer were assessed. Our results demonstrate that NE induces potent anti-HA responses in young and pregnant mice, protecting both the immunized mice and their offspring against subsequent influenza virus challenge. Furthermore, NE exhibits a dose-sparing effect on vaccine immunogenicity.

## Results

### Adjuvant effect detection

**Fig. 1:**
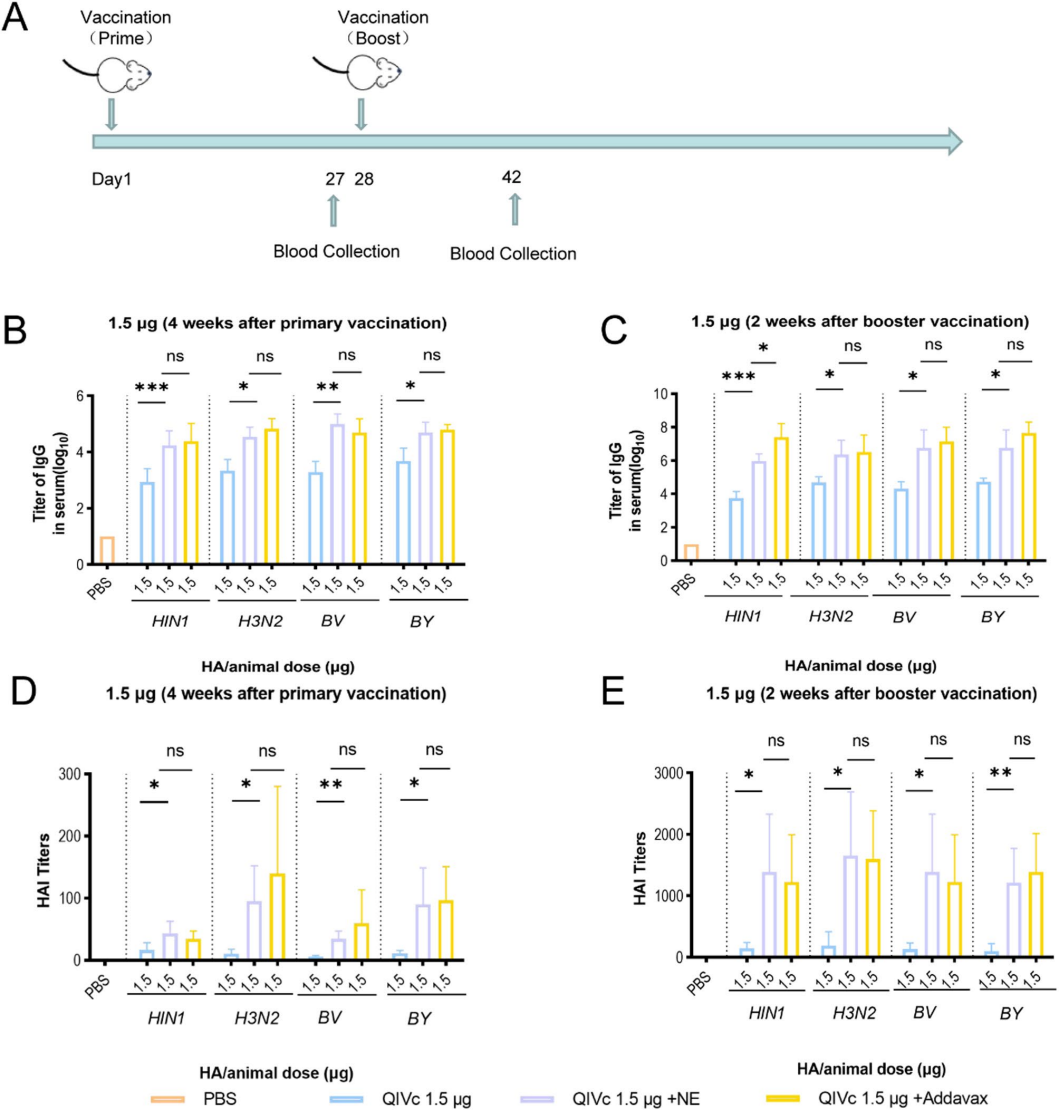
NE adjuvant as an effective adjuvant for QIVc: (**A**) Experimental diagram. BALB/c mice (*n* = 6 per group) aged 6–8 weeks were inoculated with unadjuvanted QIVc (1.5 µg HA equivalent per mouse) or QIVc combined with NE or Addavax. After 4 weeks of primary immunization, serum subtype-specific IgG levels (**B**) were determined by ELISA, and HAI titers (**D**) were measured using 4 HA units per well of each influenza virus strain. After 2 weeks of booster immunization, serum IgG levels for each strain (**C**) and HAI titers (**E**) against each influenza virus strain (4 HA units/well) were detected by ELISA. Statistical analysis was performed using one-way ANOVA (*p < 0.05, **p < 0.01, ***p < 0.001)

To evaluate the immunogenicity-enhancing effect of the nanoemulsion adjuvant, blood samples were collected from mice at 4 weeks after primary immunization and 2 weeks after booster immunization for subsequent analysis. The results showed that the total IgG titers against all four strains in the serum of the NE-adjuvanted QIVc group were significantly higher than those of the non-adjuvanted QIVc group (*p* < 0.05) (Fig. 1B and C), indicating that the adjuvant can enhance vaccine antibody titers. The NE-adjuvanted QIVc group achieved antibody levels comparable to those of the non-adjuvanted group after booster immunization with just a single primary immunization, confirming the adjuvant’s ability to enhance the vaccine’s immune response. With the exception of the 1.5 µg H1N1 strain group, no significant differences were observed between the nanoemulsion adjuvant and the commercially available squalene-based emulsion. These results demonstrate that the nanoemulsion adjuvant effectively stimulates antibody production and exhibits effects similar to those of the commercially available squalene-based emulsion.

HAI assays also showed trends similar to those of IgG antibody levels (Fig. 1D and E). For influenza A (H1N1 and H3N2), influenza B (BV and BY), the non-adjuvanted QIVc group alone can induce low levels of HAI titers, and with adjuvants, higher titers of HAI antibodies. Compared to the non-adjuvanted QIVc group, the geometric mean titers against H1N1, H3N2, and BY strains in the NE-adjuvanted QIVc group had exceeded the protective threshold (1:40) following primary immunization (Fig. 1D).After booster immunization, the anti-BY HAI titer increased the most in the NE-adjuvanted QIVc group, and the HAI titers in the NE-adjuvanted QIVc group were similar to those recorded in the Addavax-adjuvanted QIVc group (Fig. 1E). These results suggest that NE is effective in stimulating antibody production and is similar to commercially available squalene emulsions. The nanoemulsion (NE) shares a similar composition to the commercially available squalene-based adjuvant Addavax, as both are oil-in-water emulsions based on squalene. The Addavax adjuvant has a particle size ranging from 120 nm to 170 nm and a squalene content of 37–45 mg/ml. In comparison, the nanoemulsion (NE) exhibits a particle size between 145 nm and 164 nm, with a squalene content of 37–49 mg/ml. Both formulations fall within comparable ranges in terms of particle size and squalene concentration.

### Serum IgG and HAI antibody responses

**Fig. 2:**
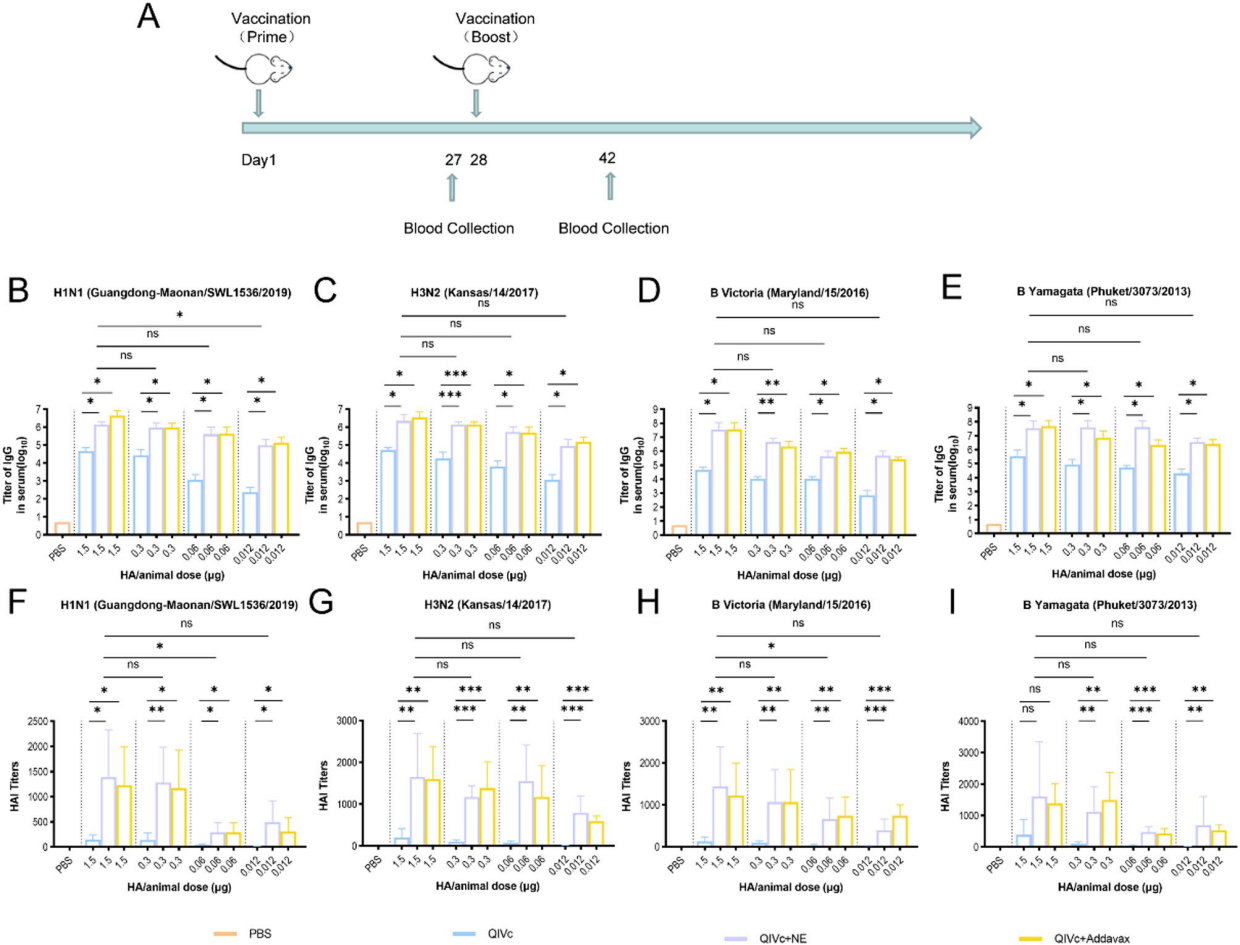
Serum IgG and HAI antibody titers in mice after booster immunization with different doses of antigen-compatible adjuvant. (**A**) Schematic representation of the experimental design. Six-to eight-week-old BALB/c mice (*n* = 6 per group) were vaccinated with unadjuvanted QIVc (1.5, 0.3, 0.06, or 0.012 µg HA equivalent per mouse) or QIVc combined with NE or Addavax adjuvant. Blood samples were collected at different time points for ELISA and HAI titer analyses. At 2 weeks after booster immunization, serum subtype-specific IgG levels (**B-E**) were determined by ELISA, and HAI titers (**F-I**) were measured in mouse sera using 4 HA units/well of influenza virus. One-way ANOVA was used for statistical analysis (*p < 0.05, **p < 0.01, ***p < 0.001)

To investigate the adjuvant activity of the nanoemulsion adjuvant in mice at different antigen doses, this study immunized BALB/c mice with varying doses of the quadrivalent influenza split vaccine (QIVc), either formulated with or without the nanoemulsion adjuvant (Fig. 2A). The humoral immune response of the vaccine was systematically evaluated by measuring serum IgG antibody titers and hemagglutination inhibition (HAI) antibody levels after booster immunization.

Regarding IgG antibody responses, no significant differences in IgG antibody titers were observed between the NE-adjuvanted and Addavax-adjuvanted QIVc groups across all tested strains (H1N1, H3N2, BV, and BY) after booster immunization. For the H1N1 strain, at all four antigen dose levels (1.5 µg, 0.3 µg, 0.06 µg, and 0.012 µg), both NE-adjuvanted and Addavax-adjuvanted QIVc groups demonstrated significantly higher antibody titers compared to the non-adjuvanted vaccine groups. Similar trends were observed for the H3N2 strain and both influenza B strains. For the NE-adjuvanted QIVc groups, comparative analysis across different antigen doses revealed significant differences only between the 1.5 µg group and the 0.012 µg group for the H1N1 strain. No statistically significant differences were observed among different antigen doses for the other tested strains (Fig. 2B, C, D and E). These results demonstrate that the incorporation of adjuvant can effectively reduce antigen usage while maintaining immunogenicity, thereby potentially lowering production costs.

In the assessment of HAI antibody levels, we compared the geometric mean titers (GMT) of homologous HAI antibodies between adjuvant-formulated groups and non-adjuvanted vaccine groups across four antigen dose levels (1.5 µg, 0.3 µg, 0.06 µg, and 0.012 µg). Following primary immunization, the HAI titer in the 1.5 µg NE-adjuvanted QIVc group exceeded the protective threshold (1:40), demonstrating that adjuvant incorporation reduces the number of immunizations required to achieve protective levels (Suppl. Figure 1). For the H1N1 strain at the 1.5 µg dose, the NE-adjuvanted group showed a 9.45-fold increase in GMT compared to the non-adjuvanted group, while the Addavax-adjuvanted group exhibited an 8.36-fold enhancement, indicating comparable potency between the two adjuvants (Fig. 2F). Across all four antigen dose levels (1.5 µg, 0.3 µg, 0.06 µg, and 0.012 µg), similar patterns were observed for both influenza A strains and both influenza B strains: both NE-adjuvanted and Addavax-adjuvanted QIVc groups demonstrated significantly higher antibody titers than the non-adjuvanted vaccine groups (Except for the BY 1.5 µg group) (Fig. 2F, G, H and I). In summary, the NE adjuvant exhibited comparable efficacy to the commercially available Addavax adjuvant in inducing both IgG antibody responses and functional HAI antibody levels against all tested influenza strains.

### Cellular immune response detection

**Fig. 3:**
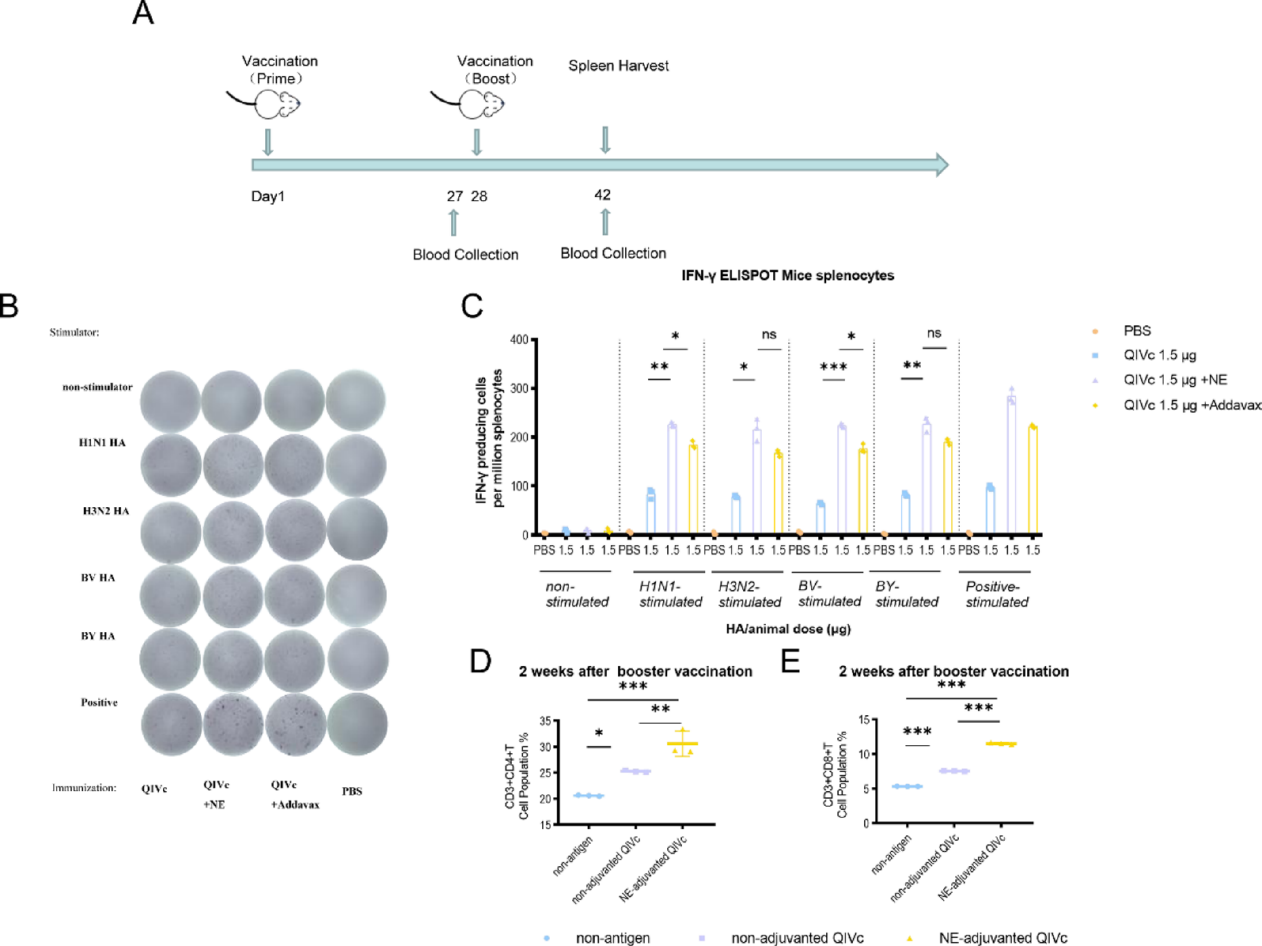
Nanoemulsion adjuvant induces T cell responses in vaccinated animals. Six- to eight-week-old BALB/c mice were vaccinated with QIVc with or without adjuvant, and spleens were harvested 14 days post-boost (**A**). T cell activation was examined by IFN-γ ELISPOT assay upon restimulation with influenza HA (**B**,** C**). Mouse spleen mononuclear cells were isolated by density gradient centrifugation on Ficoll-Paque. Flow cytometry analysis was performed to determine the levels of CD3 + CD4 + and CD3 + CD8 + T cells in immunized mice (**D**,** E**). One-way ANOVA was used for statistical analysis (*p < 0.05, **p < 0.01, ***p < 0.001).

To evaluate the adjuvant effect on T-cell immunity, IFN-γELISPOT assays were employed to compare antigen-specific cellular responses between QIVc-plus-adjuvant and QIVc-alone groups. After stimulating lymphocytes in the spleen of mice with antigen, there was a significant difference (*p* < 0.05) in the number of antigen-specific IFN-γspot-forming cells between the group receiving QIVc with adjuvant and the group receiving QIVc alone for each strain (Fig. 3B, C). To obtain more detailed information on the quality of the T cell responses observed, we further characterized the responsive T cell populations and changes in CD4 + and CD8 + T lymphocytes by flow cytometry. As shown in Fig. 3D and E, immunization with the 1.5 µg NE-adjuvanted QIVc induced higher numbers of CD4 + and CD8 + T lymphocytes in the spleen.

### Viral challenge

**Fig. 4:**
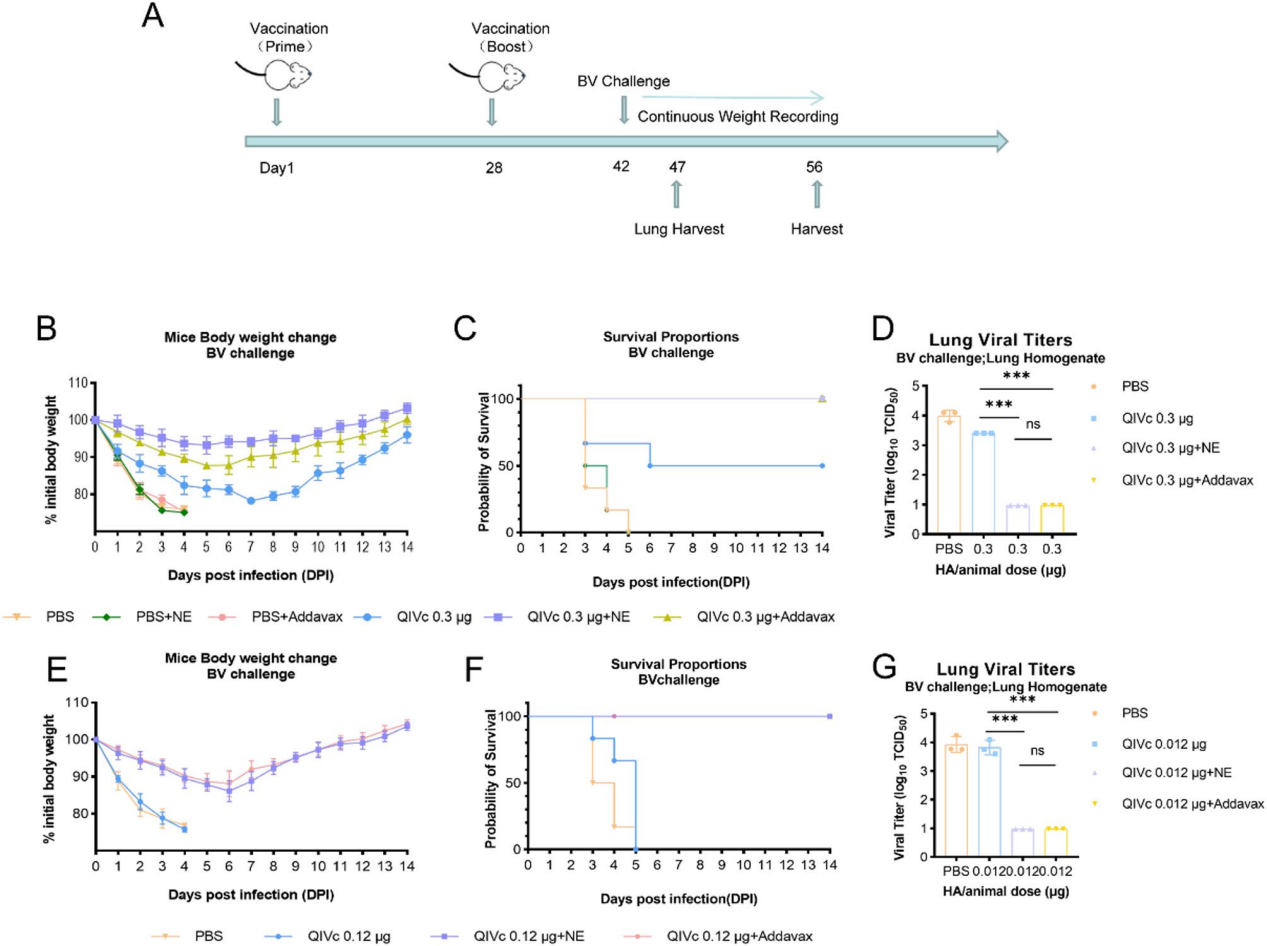
QIVc combined with adjuvant protects animals against a lethal dose of influenza virus. All unvaccinated animals and those vaccinated with 0.3 µg–0.012 µg QIVc with or without adjuvant were intranasally challenged with 10 LD₅₀ of influenza BV virus (**A**). Body weights were recorded daily for 14 days post-infection and are expressed as a percentage of initial body weight. Day 0 represents the day of infection (**B**,** E**). Survival rates were recorded daily for 14 days post-infection (**C**,** F**). Viral lung titers were quantified by TCID₅₀, with *n* = 3 animals per group (**D**,** G**). One-way ANOVA was used for statistical analysis (*p < 0.05, **p < 0.01, ***p < 0.001)

To elucidate the adjuvant-mediated enhancement of protective efficacy under low-dose antigen conditions, mice were immunized with 0.3 µg–0.012 µg formulations and subsequently challenged with a lethal dose (10 LD₅₀) of B/Victoria (Maryland/15/2016) (BV) influenza virus for protection assessment. By comparing body weight and survival rates, mice in the non-vaccinated group (PBS group) experienced rapid weight loss after infection and succumbed to illness and death by day 3. Mice in the NE-adjuvanted QIVc group showed slight weight loss in the first 5 days, with fluctuations within 10% of the initial weight, followed by a slow increase. Mice in the non-adjuvanted QIVc group reached the lowest body weight on the seventh day after infection, followed by weight gain (Fig. 4B and C). Compared to the PBS group, the lung virus titers in the QIVc adjuvant group were lower than the detection limit (Fig. 4D). The test results for the 0.012 µg NE-adjuvanted QIVc group showed a significantly enhanced protective effect against lethal virus infection, with the health status of immunized mice higher than that of the non-adjuvanted QIVc group. Mice receiving 0.012 µg non-adjuvanted QIVc alone died within 5 days, with a weight loss of over 25% (Fig. 4E). Mice receiving the 0.012 µg adjuvanted QIVc vaccine were protected from live influenza virus challenge, with significantly higher survival rates compared to the non-adjuvanted QIVc group (Fig. 4F). Compared to the non-adjuvanted QIVc group, the lung virus titers in the QIVc adjuvant group were lower than the detection limit (Fig. 4G).

### Passive maternal immunization

**Fig. 5:**
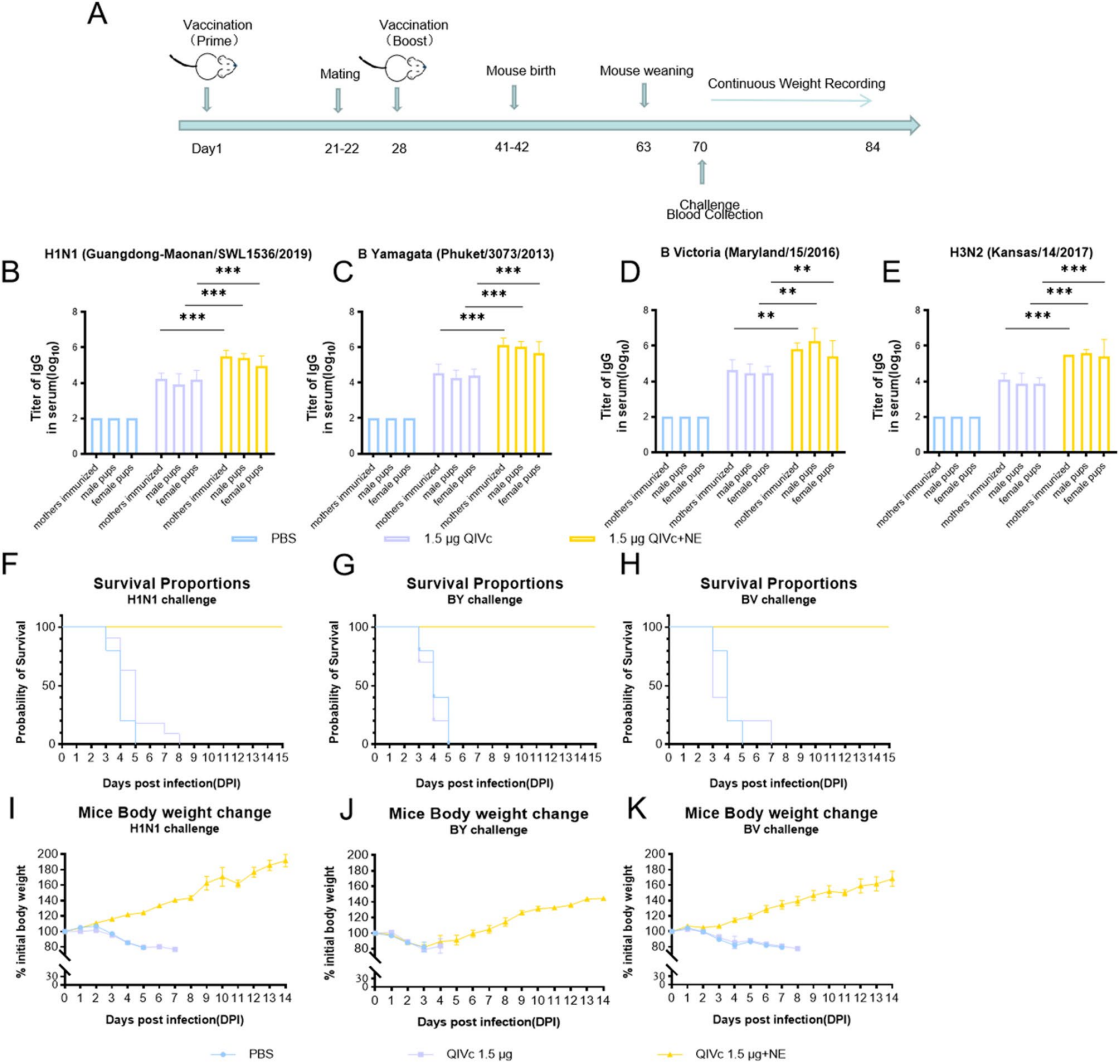
Maternal antibody transfer of anti-HA IgG in pups of immunized female mice and protection against a lethal influenza virus challenge. Female BALB/c mice (*n* = 9 per group) were immunized intramuscularly with HA protein alone (1.5 µg) or combined with NE on days 0 and 28. Pups were born on study days 41–42 and weaned 3 weeks later. Serum was collected at 4 weeks of age (study day 70) and analyzed for anti-HA IgG antibodies by ELISA (**A-E**). Body weights were recorded daily for 14 days post-infection and are expressed as a percentage of initial body weight. Day 0 represents the day of infection (**I-K**). Survival proportions were recorded daily for 14 days post-infection (**F-H**)

Pregnant women and infants are particularly susceptible to influenza-related complications and are often overrepresented in hospital admissions; therefore, we also evaluated NE in pregnant BALB/c mice and pups from immunized mothers. The transfer of maternal antibodies to the pups and the pups’ protection from influenza challenge were assessed. The female mice were mated on days 21 and 22 of the study; pups were born on days 41–42, weaned 3 weeks later, and challenged with influenza strain at 1 week post-weaning (Fig. 5F–K). To assess the transfer of maternal antibodies to the pups, serum anti-HA IgG was assessed at 4 weeks of age (70 days after their mother’s initial vaccination) (Fig. 5B-E). The antibody titers in the NE groups were significantly higher than those in the non-adjuvanted groups. Following infection with influenza strains, pups from naïve non-immunized mothers or from mothers that received QIVc alone all succumbed to infection within one week. Pups from adjuvant-vaccinated mothers exhibited 100% protection with undetectable viral titers in lung tissue. Overall, these results show that QIVc combined with adjuvant can induce potent anti-HA responses in young and pregnant mice, which can protect against subsequent viral infection.

## Discussion

Influenza vaccines remain the most effective means of preventing influenza outbreaks. However, in current mainstream production platforms utilizing chicken embryos, the emergence of adaptive mutations during cultivation can reduce vaccine efficacy [5]. As influenza vaccine research advances, evidence indicates that cell-cultured influenza viruses exhibit closer antigenic matching to circulating strains. Consequently, cell-based influenza vaccines have been approved for use in the United States, the European Union, and South Korea [12]. Building on this progress, formulating influenza vaccines by combining adjuvants with antigens appears to be a feasible and more effective strategy to induce robust immune responses against target antigens. Adjuvant incorporation has been shown to enhance vaccine efficacy across diverse population groups. Studies demonstrate that squalene-based adjuvants, compared to aluminum salts, not only improve vaccine immunogenicity and reduce antigen dosage requirements but also effectively induce cross-reactive antibodies against heterologous influenza strains [15]. In this study involving BALB/c mice, the addition of NE (nanoemulsion) to QIVc (quadrivalent inactivated influenza vaccine) resulted in a ≥ 10-fold increase in homologous IgG titers post-primary immunization across varying antigen doses, with a further ≥ 100-fold elevation following booster immunization. Squalene-based nano-adjuvants significantly amplified the magnitude of both influenza-specific IgG and hemagglutination inhibition (HI) antibody responses elicited by cell-based QIVc.

In humans, it has been shown that HA-specific IgG plays an important role in protection against influenza [16]. At high concentrations, HA-specific IgG is believed to reduce viral infection incidence, while at lower concentrations, it may prevent or alleviate disease severity after infection [17]. An interesting observation emerged when comparing changes in IgG titers across different strains induced by adjuvanted vaccines at varying doses. After booster immunization, no significant differences in IgG titers were observed for H3N2, BY, or BV strains between the 1.5 µg and 0.012 µg QIVc + NE dose groups, whereas a significant difference was detected for the H1N1 strain. This suggests that adjuvants administered at both high and low antigen doses can induce antibodies with comparable affinity, highlighting the efficiency of the BALB/c mouse immune system. Further research using different mouse strains could be conducted to investigate antibody affinity. In comparison, in human trials, the use of similar oil-in-water adjuvants, such as MF59, and a dose-saving formulation (7.5 µg + MF59) with A/California/07/2009 H1N1 split virus antigen increased seroconversion rates compared to standard non-adjuvanted vaccine (15 µg) [18].Compared to the high-dose adjuvanted vaccine (15 µg), there was a significant antigen dose-response relationship (*p* < 0.0001) in the dose-saving formulation (3.8 µg + adjuvant) using a similar oil-in-water adjuvant and A/Vietnam/1194/2004 (H5N1), but this relationship was not evident (*p* = 0.095) in the adjuvanted formulation [19].

The NE-adjuvanted, dose-sparing vaccine induced antigen-specific immune responses comparable in some aspects to those elicited by high-dose vaccines. The substantial increase in antibody titers across doses suggests the potential value of NE adjuvants for dose-sparing strategies. Following primary immunization, HI antibody titers against multiple strains in the 0.012 µg QIVc + NE group showed no significant difference compared to those in the 1.5 µg QIVc group (*p* >0.05), indicating adjuvant-mediated compensation for antigen dose reduction. Although the study did not determine the minimum effective antigen dose, the strain-dependent variations in IgG antibody titers revealed that adjuvant-mediated immunogenicity enhancement is antigen-specific. Comparable titer increases were observed in Addavax-adjuvanted vaccines, consistent with previous findings that MF59 incorporation allows drastic antigen sparing in influenza vaccines. For instance, MF59 adjuvanation enabled full protection against viral challenge with 65–80-fold lower antigen doses than unadjuvanted vaccines [20]. This dose-sparing effect holds critical implications for pandemic scenarios requiring rapid mass production of effective vaccines.

In the case of influenza vaccines, serum antibody titers measured by the Hemagglutination Inhibition (HAI) assay are widely recognized as correlates of protection. Historically, an HAI antibody titer ≥ 40 and at least a 4-fold increase in antibody titer post-vaccination have been considered immunological correlates of protection against influenza infections, associated with a 50% reduction in disease risk [21]. The Microneutralization (MN) assay demonstrates higher sensitivity than the HAI assay in detecting functional antibodies against highly pathogenic influenza viruses. However, no standardized protective threshold has been established for the MN test [22].Following primary immunization, none of the geometric mean antibody titers (GMTs) in the QIVc group exceeded 40 HAI units. In contrast, after booster immunization, HAI titers in the 1.5 µg and 0.3 µg QIVc groups surpassed this threshold (≥ 40), indicating an enhanced immune response. Notably, the 0.012 µg QIVc + NE group showed no significant differences in H1N1, H3N2, or B/Yamagata (BY) HAI antibody titers compared with the 1.5 µg antigen group after primary immunization. This suggests that the nanoemulsion adjuvant effectively compensates for reduced antigen dosage. These findings align with the IgG antibody results.

While serum antibody responses are an important determinant of vaccine efficacy, increasing evidence from cellular immune-level experiments suggests that cellular immune responses, alongside functional antibodies, also play a key role in evaluating vaccine-induced immunity, particularly in preventing severe influenza complications and death [23, 24]. Currently, the detection of interferon-gamma (IFN-γ) levels has gradually replaced various traditional cellular immune assays as the primary method for assessing cellular immune responses [25]. Immunization with the cell-based vaccine alone induced a measurable cellular immune response in mice, consistent with previous studies. The number of spleen cell spots producing antigen-specific IFN-γ was significantly different between the QIVc + adjuvant group and the QIVc group across all strains (*p* < 0.05). Post-vaccination IFN-γ production levels indirectly reflect helper T cell activity. Effective induction of T follicular helper (Tfh) cells promotes the generation of more germinal center B (GCB) cells and long-lived plasma cells, thereby prolonging neutralizing antibody persistence and broadening the neutralization spectrum [26]. However, this study did not investigate these mechanisms, and further research is required to elucidate adjuvant mechanisms and the durability of adjuvanted vaccine-induced antibodies.

The ability to confer protection against lethal viral challenge directly reflects vaccine efficacy. Assessing viral load in the lungs post-infection serves as a prognostic indicator for influenza severity and vaccine effectiveness. Heightened viral replication in the lungs correlates with intensified immune responses, resulting in exacerbated or protracted symptoms and elevated risks of multi-organ involvement. Notably, rapid and extensive viral proliferation fails to elicit a commensurate increase in pathogen-specific antibodies, thereby worsening clinical outcomes and predisposing individuals to influenza-related complications. Our findings revealed that all NE-adjuvanted QIVc formulations achieved lung viral titers below detection thresholds and significantly enhanced murine survival rates, consistent with prior reports on adjuvanted subunit influenza vaccines. Integration of serological analyses and challenge data demonstrates that NE adjuvantation enables a 125-fold antigen dose reduction while conferring multifaceted benefits: augmented humoral immune responses, suppressed pulmonary viral replication, and improved survival outcomes. These results position NE as a potent dose-sparing adjuvant for influenza vaccines.

We assessed the in vivo toxicity of the NE in female BALB/c mice. Mice receiving intramuscular injections of the NE maintained similar body weights compared to those receiving PBS injections. All mice showed a stable increase in body weight within 7 days after immunization (Suppl. Figure 2 A). No physiological symptoms such as diarrhea, lethargy, hunching posture, or disheveled fur were observed in any of the groups. The overall condition of the mice was good, with no ulceration or abscesses at the injection site, normal body temperature, and no apparent clinical abnormalities. No deaths occurred during the observation period (Suppl. Figure 2B). In both the PBS and NE groups, mice exhibited a slight decrease in body weight 2 days after immunization, followed by a slow increase in weight, with individual mice not exceeding a weight loss of 10% throughout the study. On the 7th day after muscle immunization, major organs including the heart, liver, spleen, lungs, and kidneys were collected from mice in the PBS and NE groups and stained with hematoxylin and eosin (HE) to evaluate the presence of transient tissue damage. By comparing the HE images of these organs between the two groups (Suppl. Figure 2 C), no signs of damage were observed, indicating the absence of transient tissue injury. Overall, intramuscular administration of the NE was deemed safe in the mouse model.

In conclusion, this study presents several key findings: (i) In the adult BALB/c mouse model, the nanoemulsion (NE) significantly enhanced HA-specific functional and binding antibody levels when incorporated into a quadrivalent seasonal QIVc vaccine; (ii) The NE-adjuvanted vaccine elicited antigen-specific immune responses comparable to high-dose vaccines in certain parameters, achieving a 125-fold antigen dose-sparing advantage; (iii) The adjuvant effect of NE was demonstrated through three criteria: enhanced post-immunization antibody titers, reduced viral replication in lungs post-infection, and improved survival rates.Given the challenges posed by influenza antigenic drift and suboptimal vaccine efficacy, our findings advocate for a multi-pronged prevention strategy. Through systematic experimentation, this study reveals that NE not only stimulates robust antigen-binding antibody production and protective immunity—matching the performance of commercial adjuvants—but also offers a critical antigen-sparing benefit.These results position nanoemulsion adjuvants as promising candidates for advancing influenza vaccine development, particularly in scenarios requiring dose optimization without compromising immunogenicity.

## Conclusions

When tested as an adjuvant for cell culture-based quadrivalent influenza virus subunit vaccines, intramuscular immunization with the NE-adjuvanted QIVc induced serum IgG titers approximately 10-fold higher than those of the naked QIVc. When co-administered with different doses of QIVc, NE enhanced serum IgG levels and HI titers against both IAV and IBV. The adjuvant effect was further amplified after booster immunization. In addition to enhancing humoral immunity, NE significantly improved cellular immune responses (IFN-γ production and CD8+/CD4 + T cell populations). Since clinical protection ultimately relies on post-infection outcomes (rather than immunogenicity alone), a viral challenge experiment was conducted. Results demonstrated that 100% survival could be achieved even with a low-dose immunization of QIVc (0.012 µg) combined with NE. Furthermore, preliminary data from body weight monitoring and histopathological analysis of organ tissues suggested the safety profile of NE at elevated doses in mice. It should be noted that this study has several limitations: first, all experimental data were derived from murine models, and interspecies differences in immune responses between mice and humans exist; additionally, long-term safety evaluation and data on the durability of protection remain to be established; finally, although the pregnancy animal model demonstrated maternal-fetal safety, clinical translation requires further validation.

In summary, these findings suggest, rather than prove, that NE can induce anti-hemagglutinin immune responses in adult and pregnant mice within the animal model, conferring protection against viral infection to both dams and offspring. These preliminary results indicate the potential of NE as an injectable vaccine adjuvant, though its practical application value requires confirmation through more comprehensive preclinical studies and subsequent clinical trials.

## Materials and methods

### Vaccines and viruses

The tested quadrivalent subunit influenza vaccine was developed by Changchun Institute of Biological Products Co., Ltd., containing H1N1 (Guangdong-Mao nan/SWL1536/2019), H3N2 (Kansas/14/2017), B Yamagata (Phuket/3073/2013) (BY), and B Victoria (Maryland/15/2016) (BV) influenza strains. The vaccine was 0.5 mL per dose, with no thiomersal preservative. The standard antigens and standard sera used in the experiments were purchased from the National Institute for Biological Standards and Control (NIBSC), including Influenza antigen A/Guangdong-Maonan/SWL1536/2019(CNIC1909)(H1N1)(19/312), Influenza antigen A/Kansas/14/2017(NY MC X-327)(H3N2)(19/104), Influenza antigen B/Phuket/3073/2013(B Yamagata lineage)(21/136), Influenza antigen B/Maryland/15/2016(NYMC BX-69 A)(18/104), Influenza anti-A/Guangdong-Maonan/SWL 1536/2019-like(H1N1)HA Serum(19/314), Influenza anti-A/Kansas/14/2017- Like(H3N2)HA Serum(19/152), Influenza anti-B/Phuket/3073/2013-like(B Yamagata Lineage)HA serum(19/322), Influenza anti-B/Colorado/06/2017-like HA serum(18/170). The influenza strains used in the experiments were obtained from Changchun Institute of Biological Products Co., Ltd., including H1N1 (Guangdong-Mao nan/SWL1536/2019), H3N2 (Kansas/14/2017), B Yamagata (Phuket/3073/2013) (BY), and B Victoria (Maryland/15/2016) (BV) influenza strains.

### Single radial immunodiffusion assay

The concentration of influenza virus hemagglutinin (HA) protein was quantitatively determined using the single radial immunodiffusion (SRD) assay. The H1N1, H3N2, BV, BY were treated with 1% of Zwittergent 3–14 at room temperature for at least 30 min. The standard antigen was prepared at four concentrations (40, 30, 20 and 10 µg/mL) for the standard curve. Serial dilution was performed in treated samples at four concentrations to fit the linear range of the standard. For preparing diffusion gels, the reference anti-serum was added to 1% agarose dissolved in phosphate-buffered saline (PBS) containing 0.01% Sodium azide and filled in a plastic backing. After the agarose solidified, the wells were punched and filled with 20 µL of each diluted standard antigen and test sample. The antigen–antibody reaction diffused at room temperature in a humidified chamber for 20 h and caused a zone of precipitation around the well. When the reaction was completed, the gel was washed, dried, stained by Coomassie Blue and destained by decolorizing solution. The precipitation zones were recorded and measured for the determination of HA content based on the reference standards. All samples were measured in duplicate.

### Adjuvant

The oil-in-water NE was prepared based on a previously reported method [27]. It consists of an aqueous phase (10 mM citrate buffer (5.29 mg/ml sodium citrate dihydrate and 0.34 mg/ml citric acid monohydrate) with pH between 6.0 and 6.5, 4.78 mg/ml polysorbate 80 (Tween 80), and an oil phase (43 mg/ml squalene oil and 4.78 mg/ml sorbitan trioleate (Span 85)). The aqueous and oil phases were mixed together in a high speed mixer batch mode at 10,000 rpm for 25 min. The emulsification process was performed using a high-pressure homogenizer operating at ~ 15,000 psi, with the emulsion being processed for 10 cycles until the desired particle size was obtained. The emulsion was filter-sterilized through a 0.22 μm PVDF filter using a peristaltic pump. Following filtration, the emulsion was filled at 1.5 mL volume in 7.5 mL glass vials, which were closed with polytetrafluoroethylene (PTFE)-coated chlorobutyl stoppers and aluminum caps. Following this established procedure, we subsequently prepared the NE for our studies.

### Animal studies

Female BALB/c mice (6–8 weeks old) were obtained from the Changchun Institute of Biological Products and randomized into seven groups (*n* = 6 per group). On days 0 and 28, antigens and emulsions were mixed in a 1:1 v/v ratio, and 100 µL of the vaccine was administered intramuscularly into the thigh muscle. Four weeks after the primary immunization, the same amount of adjuvant and antigen was injected into the muscle of immunized mice for booster immunization. Twenty-seven days after the first immunization and two weeks after the booster shot, blood was collected from the retro-orbital plexus and left at room temperature for 20 min. Serum was collected and stored at − 20 °C until antibody testing.

For mating, female mice were housed with male BALB/c mice for two consecutive nights at a ratio of 2 females to 1 male. Pups born to immunized female mice were weaned at 3 weeks of age and separated by sex.

For challenge tests, six mice from each group were immunized as described above and intranasally challenged with 100 µL of influenza virus suspension (10 LD₅₀) 14 days after the last vaccination. Virus challenge studies were performed under pentobarbital anesthesia (50 mg/kg, administered via thigh muscle injection). Animals were euthanized by cervical dislocation 5 days after challenge, and the lungs were harvested. The left lung lobe was homogenized and used for virus titration.

All mice in this study were handled in accordance with the Guide for the Care and Use of Laboratory Animals (National Research Council). All experimental procedures were reviewed and approved by the Animal Welfare and Research Ethics Committee of the Changchun Institute of Biological Products.

### Serum IgG assay

Antibody titers were detected using enzyme-linked immunosorbent assay (ELISA). First, 96-well plates were coated with 0.10 µg influenza HA protein per well and incubated overnight at 4 °C. The plates were then blocked with 1% bovine serum albumin in PBS overnight at 4 °C. After washing with PBS containing 0.05% Tween-80, threefold serially diluted serum samples were added to the wells and incubated at 37 °C for 1 h. Antigen-specific antibody levels in serum samples were detected using anti-mouse HRP-IgG, while total IgG antibody levels were determined with anti-goat HRP-IgG (ZSbio, China; dilution ratio 1:6000). Both secondary antibodies were incubated at 37 °C for 1 h. Subsequently, 100 µL of 3,3′,5,5′-tetramethylbenzidine (TMB) substrate was added to each well, and the plates were incubated at 37 °C in the dark for 20 min. The reaction was terminated by adding 2 M HCl. Absorbance was measured at 450 nm and 630 nm. Antibody titers were defined as the highest dilution at which the absorbance value was twofold greater than the average OD of the blank control. Geometric mean titers for each group are presented with standard error of the mean (SEM).

### HI assay

Prior to hemagglutination inhibition (HI) testing, fresh chicken erythrocytes were washed three times with phosphate-buffered saline (PBS, pH 7.4) using 15 mL centrifuge tubes (centrifuged at 1500 rpm for 5 min each time). Packed chicken erythrocytes and a 1.0% chicken erythrocyte suspension were then prepared in PBS. All serum samples were pretreated with receptor-destroying enzyme (RDE; Denka Seiken, Japan) at 37 °C for 18 h to eliminate non-specific inhibitors, followed by treatment with packed chicken erythrocytes at 4 °C for 1 h to remove natural serum agglutinins. Serum samples were subjected to two-fold serial dilution in 96-well V-bottom plates using PBS. An equal volume of standardized influenza virus hemagglutinin antigen (4 HAU/25 µL) was added to each well to facilitate binding between HA and serum anti-HA antibodies. The serum-virus mixture was incubated at room temperature for 40 min, after which 25 µL of 1% erythrocyte suspension was added to each well and allowed to settle at room temperature for 30 min. The plates were then tilted to observe hemagglutination inhibition units, and all agglutination patterns were read within 45 min. Antibody titers were expressed as the reciprocal of the highest serum dilution that showed complete inhibition of hemagglutination. Seroconversion was defined as either a pre-vaccination titer < 1:8 with a post-vaccination titer ≥ 1:40, or a pre-vaccination titer ≥ 1:8 with at least a four-fold increase in post-vaccination titer.

### Elispots

Spleens were aseptically harvested from mice two weeks after the final immunization, washed with sterile PBS, and collected in RPMI 1640 medium supplemented with 10% fetal bovine serum (FBS) and 1× penicillin/streptomycin. Single-cell splenocyte suspensions were prepared by pressing the spleens through a 70 μm cell strainer. The splenocytes were collected by centrifugation at 1200 rpm. Erythrocytes were lysed using 155 mM NH₄Cl and 17 mM Tris-HCl (pH 7.2) for 1 min. Freshly isolated mouse splenocytes were seeded at a density of 1 × 10⁵ cells per well in mouse IFN-γ pre-coated ELISpot plates (Dakewe Biotech Co., Ltd., China). The splenocytes were left unstimulated or were restimulated with 10 µg/mL hemagglutinin (HA) and incubated for 18 h at 37 °C under 5% CO₂. Following incubation, the cells were removed, and the plates were thoroughly washed with washing buffer. The wells were then incubated with 100 µL of biotinylated polyclonal detection antibody against IFN-γ for 2 h at room temperature. After five washes with washing buffer, the plates were incubated with streptavidin–HRP conjugate for 1 h at room temperature. The plates were washed five more times and then incubated with substrate in the dark at room temperature. Finally, the plates were thoroughly rinsed under tap water and air-dried overnight in the dark. Spots were enumerated within 7 days using an automated ELISpot reader (AID GmbH, Strassberg, Germany) and manually verified. Results are expressed as the number of IFN-γ-producing splenocytes per million cells.

### Lung viral titers

Lungs were harvested on day 5 after influenza virus challenge. Viral titers in the lungs were determined as previously described. Briefly, the left lungs were collected in serum-free RPMI 1640 medium and homogenized using a tissue grinder. The homogenates were centrifuged at 3000 rpm for 10 min at 4 °C. The resulting supernatants were collected, stored at − 80 °C, and used for subsequent viral titer assays. The samples were subjected to twofold serial dilutions in serum-free Dulbecco’s Modified Eagle Medium (DMEM) and incubated with MDCK cells for 2 h at 37 °C. After incubation, the supernatant was removed and replaced with fresh serum-free DMEM for continued culture. On day 3, a hemagglutination assay was performed using 1% chicken red blood cells to evaluate viral titers in each lung homogenate. Viral lung titers were determined by inoculating 10-fold serial dilutions of the tissue supernatants onto MDCK cells in 96-well plates and assessing infectivity after 72 h of incubation. Viral titers were calculated using the Reed and Muench method.

### Analysis of CD4/CD8 population by flow cytometry of the splenocytes

Splenocytes were analyzed by flow cytometry using a standard staining protocol. The following fluorescently conjugated antibodies were used: FITC-anti-CD4, APC-anti-CD8, and PE-anti-CD3 (all from BioLegend, San Diego, CA, USA). Predetermined optimal concentrations of these antibodies against CD3, CD4, and CD8 were added to 0.1 mL of splenocyte suspension and incubated for 30 min on ice. Data were acquired on a BD flow cytometer and analyzed with the corresponding software.

### Statistical analysis

The statistical analysis was performed using GraphPad Prism version 8 (GraphPad Software, San Diego, CA, USA). All data were analyzed by one-way analysis of variance (ANOVA) followed by Dunnett’s test and are presented as mean ± SEM. Differences were considered statistically significant at *p* < 0.05.

## Supplementary Information

Supplementary Material 1.

## Data Availability

The datasets used during the current study are available from the corresponding author on reasonable request.
