# Supplementary material for: The nanoemulsion adjuvant provides antigen dose-sparing effects and enhances maternal passive immune protection for the cell-cultured quadrivalent influenza virus subunit vaccine
Source: Virol J. 2026 Jan 30;23:32. doi: 10.1186/s12985-025-03006-z (PMC12874696; doi:10.1186/s12985-025-03006-z)

Figure S1 Serum IgG and HAI antibody titers in mice after primary immunization with different doses of antigen-compatible adjuvant. (A) Schematic representation of the experimental design. 6-8-week-old BALB/c mice (n=6 per group) were vaccinated with unadjuvanted QIVc (1.5, 0.3, 0.06, or 0.012 μg HA equivalent per mouse) or QIVc combined with NE or Addavax adjuvant. Blood samples were collected at different time points for ELISA and HAI titer analyses. At 4 weeks after primary immunization, serum subtype-specific IgG levels (B-E) were determined by ELISA, and HAI titers (F-I) were measured in mouse sera using 4 HA units/well of influenza virus. One-way ANOVA was used for statistical analysis (*p < 0.05,**P < 0.01, ***p < 0.001,****p＜0.0001).


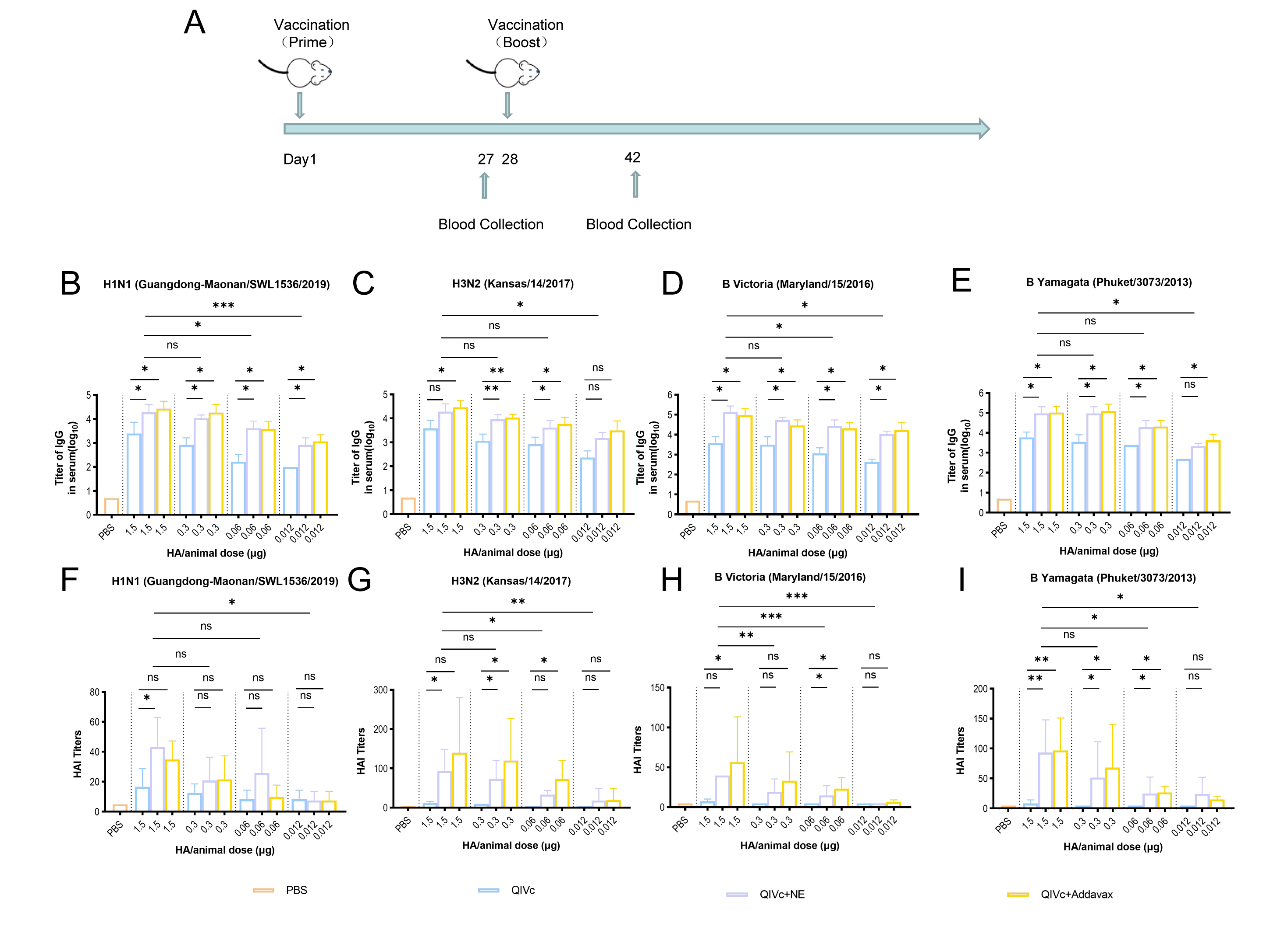


Figure S2 Safety evaluation of NE. (A) Body weight of mice in each group within 7 days after immunization (n=3). (B) The survival proportions were recorded every day for 7 days post-immunization. (C) Representative H&E-stained tissues of the mice on day 7 post-intramuscularly immunization. (Scale bar, 50 μm).


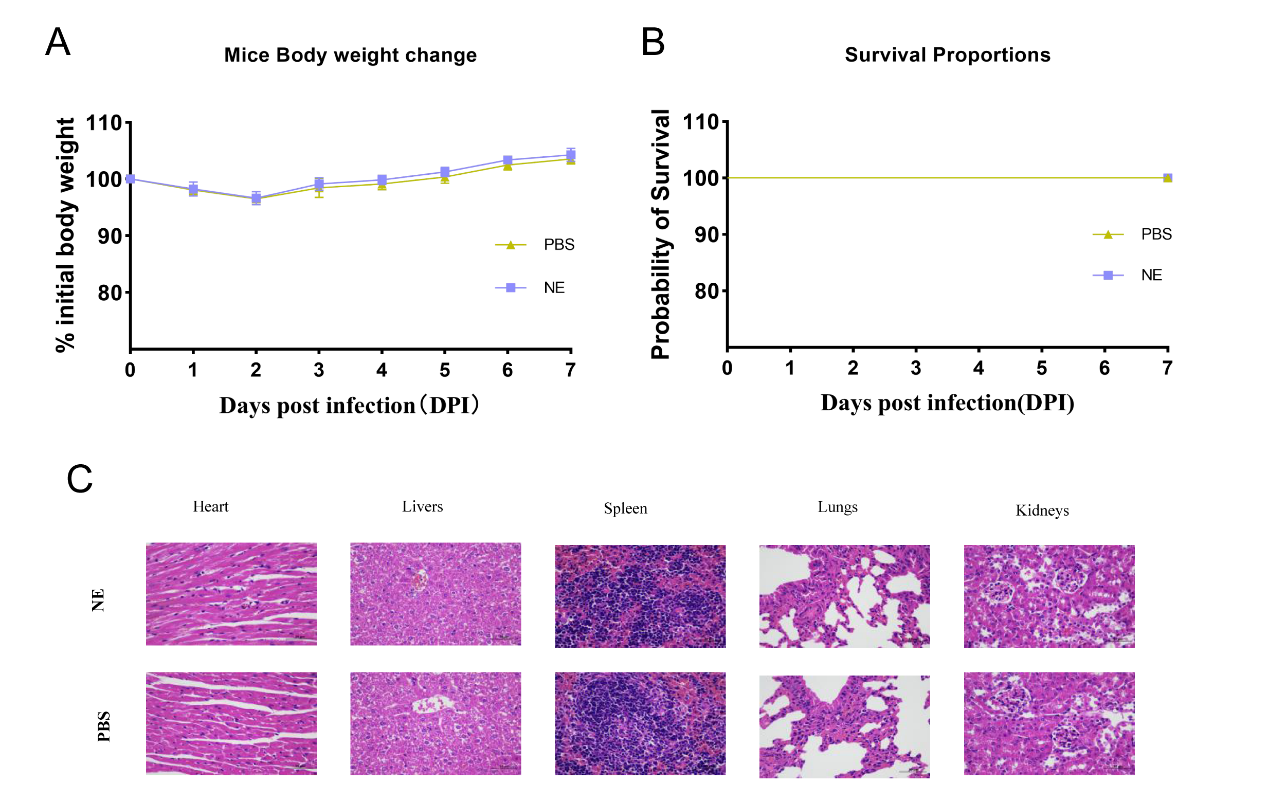

Supplement: Supplementary file 1 — Supplementary Material 1. [file 12985_2025_3006_MOESM1_ESM.docx]
